# Process development of human multipotent stromal cell microcarrier culture using an automated high‐throughput microbioreactor

**DOI:** 10.1002/bit.26359

**Published:** 2017-07-27

**Authors:** Qasim A. Rafiq, Mariana P. Hanga, Thomas R. J. Heathman, Karen Coopman, Alvin W. Nienow, David J. Williams, Christopher J. Hewitt

**Affiliations:** ^1^ Department of Biochemical Engineering, Advanced Centre for Biochemical Engineering University College London Gower Street London United Kingdom; ^2^ Aston Medical Research Institute, School of Life and Health Sciences Aston University Aston Triangle Birmingham B4 7ET United Kingdom; ^3^ Centre for Biological Engineering Loughborough University Leicestershire LE11 3TU United Kingdom; ^4^ PCT A Hitachi Group Company Allendale New Jersey; ^5^ School of Chemical Engineering University of Birmingham Edgbaston Birmingham United Kingdom

**Keywords:** human mesenchymal multipotent stromal cell, bioprocessing, microcarrier, microbioreactor, cell therapy, scale down

## Abstract

Microbioreactors play a critical role in process development as they reduce reagent requirements and can facilitate high‐throughput screening of process parameters and culture conditions. Here, we have demonstrated and explained in detail, for the first time, the amenability of the automated ambr15 cell culture microbioreactor system for the development of scalable adherent human mesenchymal multipotent stromal/stem cell (hMSC) microcarrier culture processes. This was achieved by first improving suspension and mixing of the microcarriers and then improving cell attachment thereby reducing the initial growth lag phase. The latter was achieved by using only 50% of the final working volume of medium for the first 24 h and using an intermittent agitation strategy. These changes resulted in >150% increase in viable cell density after 24 h compared to the original process (no agitation for 24 h and 100% working volume). Using the same methodology as in the ambr15, similar improvements were obtained with larger scale spinner flask studies. Finally, this improved bioprocess methodology based on a serum‐based medium was applied to a serum‐free process in the ambr15, resulting in >250% increase in yield compared to the serum‐based process. At both scales, the agitation used during culture was the minimum required for microcarrier suspension, N_JS_. The use of the ambr15, with its improved control compared to the spinner flask, reduced the coefficient of variation on viable cell density in the serum containing medium from 7.65% to 4.08%, and the switch to serum free further reduced these to 1.06–0.54%, respectively. The combination of both serum‐free and automated processing improved the reproducibility more than 10‐fold compared to the serum‐based, manual spinner flask process. The findings of this study demonstrate that the ambr15 microbioreactor is an effective tool for bioprocess development of hMSC microcarrier cultures and that a combination of serum‐free medium, control, and automation improves both process yield and consistency. Biotechnol. Bioeng. 2017;114: 2253–2266. © 2017 The Authors. Biotechnology and Bioengineering Published by Wiley Periodicals, Inc.

## Introduction

Human mesenchymal multipotent stromal/stem cells (hMSCs) are considered a promising candidate for a cell‐based therapies given their propensity for growth in vitro, relative ease of isolation, differentiation potential and their ability to secrete small molecules which can aid the regeneration of damaged tissue (Aggarwal and Pittenger, 2005). However the translation of this promising research to clinical adoption will require, among other factors, the successful development of scalable, sustainable, robust, and consistent cell manufacturing processes (Rafiq and Hewitt, 2015). There is a commercial and clinical need to expedite cell therapy process development; small‐scale, high‐throughput platforms provide a means to achieve this. Such technologies can improve efficiency, reduce costs, and accelerate time to market while minimizing development resources (Bareither and Pollard, 2011; Pollard, 2014; Rafiq and Hewitt, 2015). Moreover, the high‐throughput nature of these technologies are amenable for Quality by Design (QbD) tools such as factorial design of experiments (DoE) which have become an integral part of modern process development and manufacture.

It is well established that multifactorial statistical experimentation is necessary to identify and understand the complex interaction between key variables and parameters to develop optimal cell culture conditions which maintain product quality attributes (Mitchell et al., 2014). Multiple small‐scale, high‐throughput cell culture platforms have been developed to enable this type of experimentation, including spinner and shake flasks (ranging in minimum working volume from 50 to 250 mL), bench‐top bioreactors (ranging in volume from 250 mL to 5 L) and more recently, microbioreactors. The latter term is a general one, covering multiple types of devices (ranging in volume from 500 μL to 30 mL) providing a range of scales and complexity including microtiter plates to parallel arrays of fully monitored, controlled, and automated miniature bioreactors (Hsu et al., 2012; Nienow et al., 2013; Warr SRC, 2014).

For hMSC process development, the majority of the small‐scale work has been conducted in spinner flasks (Bardy et al., 2013; Dos Santos et al., 2011a; Eibes et al., 2010; Ferrari et al., 2012; Goh et al., 2013; Hewitt et al., 2011; Rafiq et al., 2013a; Schirmaier et al., 2014; Schop et al., 2010). Although spinner flasks are easy to use and require little training, these systems are restricted to surface aeration and are limited with respect to experimental throughput. They also do not provide an environmental control capability as found in traditional benchtop bioreactors and are dependent on external control of humidity, temperature, and oxygen concentration which is usually achieved by being placed within an incubator (Hsu et al., 2012; Jossen et al., 2014), which can have a significant laboratory footprint. In addition, each vessel has to be manipulated individually and manually with regard to medium exchange, which for many vessels takes significant time often outside the controlled environment of the incubator. To facilitate translation to the clinic, relevant, accurate, small‐scale, high‐throughput experimental models need to be developed which are representative of larger‐scale, industrial systems that will eventually be used for product manufacture.

**Figure 1:**
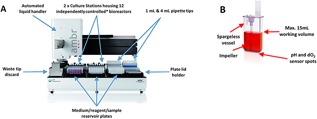
Overview of the ambr15 microbioreactor. (A) The ambr15 automated platform including liquid handler and culture stations and (B) the ambr15 microbioreactor spargeless vessel.

To address the need for small‐scale, high‐throughput cell culture technology, numerous systems have been developed including microtiter plates (Legmann et al., 2009), microfluidic reactors (Zanzotto et al., 2004) and small‐scale automated bioreactors such as the ambr15 cell culture system (Lewis et al., 2010). The ambr15 system is an automated, high‐throughput bioreactor platform which allows for 24 or 48 individually controlled, single‐use stirred‐tank bioreactors (Fig. 1). Each bioreactor has automated online monitoring and control for pH and dissolved oxygen (dO_2_). The ambr15 platform has demonstrated significant success for biologics production, where it was found to be equivalent with respect to cell growth and protein titre with larger scale stirred systems (Hsu et al., 2012; Lewis et al., 2010; Nienow et al., 2013). However, all applications of the ambr15 system thus far have focused exclusively on free suspension culture and yet many cell therapy candidates including hMSCs are anchorage‐dependent. As such, the expansion of these cells in stirred‐tank bioreactors, in most cases, requires the use of microcarriers.

The aim of the work presented here is to show that the ambr15 microbioreactor system is a suitable scale‐down model for larger scale hMSC microcarrier culture. Since, microcarrier suspension is considered critical for successful culture (Hewitt et al., 2011) and the shape of the ambr15 (rectangular cuboid of aspect ratio >1 (Nienow et al., 2013), Fig. 1) is not optimal for suspension, it was expected that modifications would be necessary; and this proved to be so. These changes are therefore discussed first, leading to significant improvements in performance. Once established, the modified ambr15 system and microbioreactors were then used for bioprocess development, whereby studies were conducted to optimize hMSC microcarrier culture conditions resulting in improved cell yields. These findings were then validated in larger‐scale vessels demonstrating equivalent cell growth, viability, identity, and functionality.

## Materials and Methods

### hMSC Monolayer Expansion

Human MSCs from two donors were isolated from bone‐marrow aspirate obtained by Lonza (Walkersville, MD) after the donor provided informed consent. The local Ethical Committee approved the use of the samples for research. The hMSCs were isolated on the basis of plastic adherence and cryopreserved at passage 1 at a density of 2 × 10^6^ cells/mL in 10% dimethyl sulphoxide (DMSO) (v/v) (Sigma–Aldrich, Dorset, UK) and 90% foetal bovine serum (FBS; Hyclone, Lot# RUF35869). To expand hMSCs for microcarrier experiments, hMSCs were cultured in monolayer as described in Rafiq et al. (2013b). In brief, the hMSCs were seeded at 5,000 cells/cm^2^ and cultured in DMEM (Lonza, Slough, UK) supplemented with 10% (v/v) foetal bovine serum (FBS; HyClone) and 2 mM UltraGlutamine (Lonza, UK). Where cells were cultured under serum‐free medium (SFM) conditions, the Prime‐XV™ SFM hMSC medium was used (Irvine Scientific, Santa Ana, CA) in accordance with the manufacturer's instructions. As required, attachment surfaces were pre‐coated with recombinant fibronectin (Irvine Scientific) and the hMSCs underwent one adaptation passage in medium containing SFM Prime‐XV™ medium. Viable cell number (via acridine orange uptake and DAPI exclusion) and mean cell diameter were determined using a NucleoCounter NC‐3000 automated mammalian cell counter (Chemometec, Lillerød, Denmark).

### Spinner Flask Microcarrier Culture

A 100 mL BellCo spinner flasks (Bellco Glass Inc., Vineland, NJ), were used for all spinner flask experiments, with a 100 mL working volume and a vessel diameter (T) of 60 mM. For all microcarrier cultures, Plastic P102‐L microcarriers (Pall Life Sciences, Portsmouth, UK) were employed (Rafiq et al., 2016). The same inoculation concentrations and culture methods were employed as described in Rafiq et al. (2016) except where improvements to the process were made (discussed in the Results section). In brief, this method employed 5 cm^2^ of microcarrier surface area per mL of medium and an inoculation density of 6,000 cells/cm^2^. The spinner flasks were agitated at the just suspended speed (N_JS_) which was 30 rpm. When additional microcarriers were added as described in later experiments N_JS_ increased to 45 rpm.

### ambr15 Microcarrier Culture

Prior to inoculation, ambr15 vessels with up‐pumping impellers were loaded onto the platform and stabilized with respect to pH (7.2), temperature (37 °C) and dO_2_ (100%). The original working volume was set to 15 mL and the original impeller speed for N_JS_ was 300 rpm. This speed was subsequently increased to 400 rpm (as described later) due to clump formation, preventing effective suspension of the microcarriers as the culture progressed. The hMSCs were cultured at the same seeding and microcarrier concentration as the spinner flasks and cultured in complete growth medium. The liquid handling program script was amended to enable the system to sample the vessel at a user‐set volume rather than the standard factory setting which would sample from the bottom of the vessel.

As with spinner flask culture, daily samples of the microcarrier culture were taken throughout culture for cell viability and supernatant analysis. Cells were counted while still attached to the microcarriers using the NC‐3000 NucleoCounter (Chemometec) as directed by the manufacturer. Two approaches for sampling were adopted during the course of this study. First, 250 μL of the microcarrier carrier was sampled daily as the vessels were agitated, thus ensuring that a representative sample of the microcarrier culture was obtained. The volume removed was replaced when a medium exchange was performed. Additional studies were performed where entire vessels were sacrificed at each time point. This procedure was conducted to identify whether the daily removal of a 250 μL sample and replacement of medium during a medium exchange had any effect on the culture. It was found that the daily sampling method did not have any notable effect on the culture (data not shown).

As described in Achieving equivalent hMSC growth in ambr15 and spinner flasks section, a strategy employed to improve culture in the ambr150 vessel involved aseptic siliconization of the vessel. This process was achieved by using a 0.2 μM syringe filter (Fisher Scientific, Loughborough, UK) and manually adding the siliconizing agent, Sigmacote (Sigma–Aldrich), to each vessel ensuring coverage of the entire vessel surface area before aspirating. Vessels were left overnight to dry in a fume hood and rinsed with distilled water after 24 h.

To achieve the higher cell densities described in Serum‐free expansion of hMSCs in ambr15 and spinner flasks section, additional microcarriers were added to the vessels during the exponential phase of growth when the cells were reaching densities ∼75,000 cells/cm^2^, a value which we have previously found to be ∼80% confluence in T‐flasks for Prime‐XV SFM cultures. Microcarrier addition was based on the intention of achieving a mean ratio of 50 cells/microcarrier and was calculated based on the cell number in each vessel and the number of microcarriers in each vessel at the point of addition.

### Cell Characterization

Multi‐parameter immunophenotypic analysis of the hMSCs was determined by flow cytometry and the antibodies were selected on the basis of the panel recommended by the International Society for Cellular Therapy (ISCT) (Dominici et al., 2006). This was performed using a Guava HT flow cytometer with excitation at 488 nM and is described in detail in Chan et al. (2014). Tri‐lineage differentiation potential of the cells was determined by using the StemPro Adipogenesis kit, StemPro Chondrogenesis kit and StemPro Osteogenesis kit (Life Technologies, ThermoFisher Scientific Company, Loughborough, UK) in accordance with the manufacturer's instructions on post‐harvest hMSC samples to induce differentiation. Staining of the samples to determine differentiation was performed as described in (Rafiq et al., 2013b). CFU‐f efficiency was determined by culturing the hMSCs in monolayer at a cell density of 10 cells/cm^2^ and with a complete medium exchange every 4 days. After 15 days in culture, hMSCs were washed with PBS (Lonza, Visp, Switzerland) and fixed with 4% formaldehyde (v/v) (Sigma, UK) for 30 min. A 1% crystal violet (Sigma–Aldrich) in 100% methanol (w/v) was used for colony staining. The samples were incubated at room temperature for 30 min in the 1% crystal violet solution. All stained colonies that comprised of more than 25 cells were recorded as CFUs.

### Harvesting of hMSCs in Spinner Flasks

A detailed overview of the harvesting method and theoretical principles are provided in (Nienow et al., 2014). Briefly, the harvesting procedure involved a two‐step process: (1) detachment of the cells from the microcarriers using an increased agitation rate of 150 rpm for 7 min during enzymatic reagent exposure and (2) separation of the cells and microcarriers prior to cell concentration by filtration. After detachment and separation, >95% cells with >95% viability were recovered (Nienow et al., 2016).

### Harvesting of hMSCs in ambr15 Microbioreactor

The harvest procedure for the ambr15 was similar in principle to that for the spinner flask harvest (Nienow et al., 2016). However, the harvest was automated and performed by the device's liquid handler. Given the time taken by the liquid handler to aspirate spent medium from each vessel (approximately 3 min/vessel) and the fact that 12 bioreactor vessels are operated on one culture station (and therefore limited to one speed for all 12 bioreactor vessels), only two vessels on each culture station were harvested at a time. This limit was imposed in order to avoid prolonged exposure to trypsin which can result in cell damage during extended waiting time. The agitation rate for harvesting was originally 650 rpm for 7 min during enzymatic reagent exposure (6 mL of either trypsin or TryplE was used—each were equally effective (Nienow et al., 2016)). However, as discussed later, the speed was subsequently increased to 800 rpm to facilitate cell harvest from clumps formed during culture. In that case, post filtration, more than 95% viable cells were recovered (Nienow et al., 2016).

### Analytical Techniques

Analysis of glucose and lactate concentrations in the spent medium was performed using a Cedex Bio‐HT (Roche, Mannheim, Germany). The specific growth rate, cumulative population doublings, specific metabolite consumption/production and coefficient of variation (CV) were calculated for all samples (Rafiq et al., 2013b).

### Statistical Analysis

For comparison between two data sets, statistical significance was determined by using the Student's two‐tailed *t*‐test. For comparison of multiple data sets, significance was calculated by the one‐way ANOVA test with the Tukey post hoc correction test; significance was determined at *P* < 0.05. GraphPad Prism 6 (La Jolla, CA) was used for all statistical analysis.

## Results

The objective of this work was two‐fold: (1) to determine whether the ambr15 microbioreactor, designed for free suspension culture, could be adapted for small‐scale, microcarrier culture; and (2), if so, could the ambr15 platform then be used for serum‐free bioprocess development, which could be reproduced in a larger scale vessel.

### Initial Adaptation of the ambr15 Microbioreactor Geometry and Software for Microcarrier Culture

As hMSCs are anchorage‐dependent and are traditionally grown on microcarriers when cultured in stirred‐tank bioreactors, it was first necessary to adapt the system to enable microcarrier culture to be undertaken successfully. This involved two key changes: (1) since the original ambr15 has a sparger which extends close to the base of the bioreactor, a spargeless vessel was used to prevent blockage and backflow of microcarriers into the sparger (Fig. 1); and (2) amendments to the liquid handler software script to enable customizable liquid sampling points for medium exchange and sampling without removing microcarriers (Fig. 1).

### Initial hMSC Microcarrier Studies in the Modified ambr15 Microbioreactor

**Figure 2:**
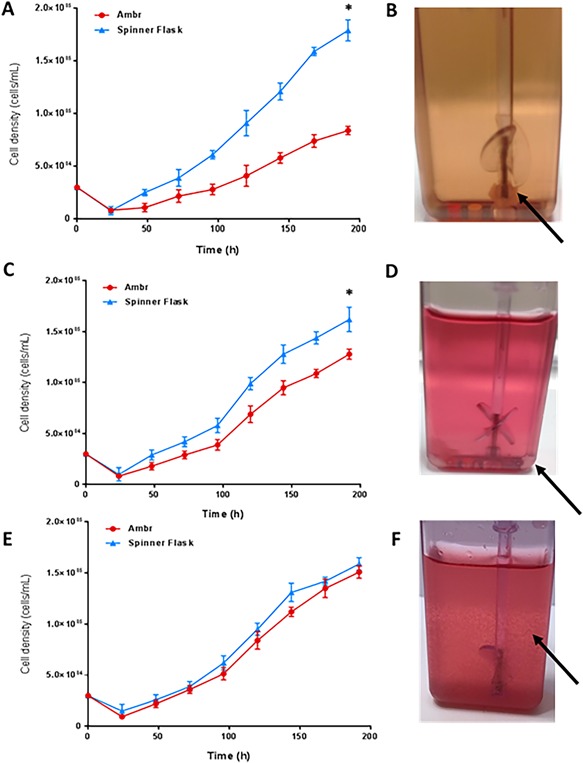
Growth of hMSCs from donor 1 cultured on microcarriers in serum‐based medium in the ambr15 and spinner flasks with (A) the viable cell density in the initial run where clumping around the ambr15 vessel impeller was a significant issue (B), the arrow illustrates microcarrier‐cell clump formation around the base of the ambr15 impeller shaft. (C) The viable cell density following changes to the agitator configuration (from up‐pumping to down‐pumping) and an increase in harvest agitation rate from 650 to 800 rpm which encouraged cell detachment from larger microcarrier clumps. (D) Reduced clumping around the ambr15 impeller shaft but clumping in corners of the ambr15 vessel illustrated by the arrow. (E) Viable cell density after further changes to avoid clumping including increasing the culture agitation rate from 300 to 400 rpm and aseptically siliconizing the vessel prior to use. (F) Improved microcarrier suspension as indicated by the arrow with little/no clumping. Data show mean ± SD, *n* = 8. (*) Significance was determined at *P* < 0.05.

Human MSCs were cultured on microcarriers in both the ambr15 microbioreactor and spinner flasks with an equivalent surface area of microcarriers to volume of medium ratio provided in both systems. Growth in the spinner flask was significantly better throughout the course of the culture, with a final viable cell density of 1.78 × 10^5^ ± 0.26 cells/mL being achieved by 192 h in culture compared with 0.73 × 10^5^ ± 0.09 cells/mL in the ambr15 (Fig. 2). After completion of the culture, the vessels were visually inspected and it was found that the vast majority of microcarriers were clumped around the base of the impeller (Fig. 2B). This large microcarrier clump was not extracted during the harvest procedure. Therefore any cells growing within this clump were unlikely to have been included in the count resulting in a low and unrepresentative number.

### Strategies to Avoid Clumping Around the Base of Impeller

Strategies were considered as to how the extensive clumping around the base of the impeller (Fig. 2B) could be avoided; and also, if any clumps were to form, how the cells could be successfully harvested. First, it was decided that a three‐fold approach would be implemented involving a change in agitation strategy. The agitator configuration was changed from up‐pumping to down‐pumping, a preferred mode for solid suspension without sparging (Nienow, 1997b) . Additionally, the agitation of the culture was stopped every 6 h for 1 min over the first 24 h which prevented microcarrier clumps forming around the base of the impeller. Finally, the agitation rate during harvest was increased from 650 rpm to 800 rpm in order to increase the harvest of cells from any microcarrier clumps that formed.

With this change in the process, there was no noticeable clumping around the base of the impeller (Fig. 2D) and all the cells on microcarriers in the vessel were extracted for harvest and counted. This change significantly improved the cell density achieved in the ambr15 with a final viable cell density of ∼1.20 × 10^5^ cells/mL by 192 h being recorded. Although the ambr15 demonstrated a similar trend in the growth compared to the spinner flask, there was still a significantly lower final viable cell density achieved in the former. After examination, it was noted that clumping was still an issue. Instead of a large clump forming around the base of the impeller as previously (Fig. 2B), clumps of microcarriers (albeit smaller in size) were now forming in the corners of the microbioreactor vessel (Fig. 2D).

### Achieving Equivalent hMSC Growth in ambr15 and Spinner Flasks

Microcarriers clumping in the corners of the ambr15 vessel suggested that increased agitation intensity was required not only during harvest but also during culture so it was increased from 300 rpm to 400 rpm. This speed was selected as it facilitated suspension of microcarriers for the duration of the culture period. In addition to further prevent microcarriers/cell clumps forming in the vessel corners, the internal surfaces of the microbioreactor and impeller were aseptically siliconized.

This combination of methods significantly improved the microcarrier suspension with them being well suspended throughout the course of the culture with significantly less clumping (Fig. 2F). As a result, the hMSC final viable cell density achieved in the ambr15 culture was 1.51 ± 0.06 cells/mL by 192 h (Fig. 2E) which was similar to that of the spinner flask value of 1.59 × 10^5^ ± 0.06 cells/mL.

### Bioprocess Development Using the ambr15 Microbioreactor System

The other key aim was to determine whether the system could be used for improved bioprocess performance and if this improvement could be validated in a larger‐scale system. We identified that a critical aspect of the culture which required optimization was the inoculation and initial cell attachment period. The growth curves in Figure 2E show a consistent, significant decrease in cell number post‐inoculation over the first 24 h for both ambr15 and spinner flasks, which results in an extended lag phase in both systems. Following investigation of a range of factors, it was found that two parameters had the most significant impact in reducing the lag period: the initial medium volume (volume of medium at the start of the culture) and intermittent agitation over the first 24 h. The adjustments in the two parameters formed what is subsequently referred to as the “improved culture process protocol.” This improved process differed from the original one in that the initial working volume was reduced by 50% (7.5 mL in the ambr15) for the first 24 h before being increased to the maximum working volume of 15 mL. The improved process also involved an intermittent agitation regime which consisted of 5 min agitation followed by 25 min without agitation for the first 3 h of culture—after this time, the culture was agitated continuously.

**Figure 3:**
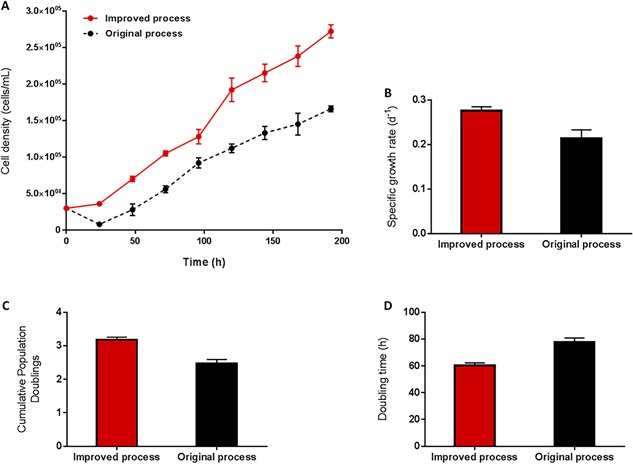
Comparison of hMSC donor 1 growth kinetics in ambr15 with the original and improved process cultured on microcarriers in serum‐based medium, showing (A) the viable cell density, (B) specific growth rate, (C) the cumulative population doublings and (D) the doubling time. Data show mean ± SD, *n* = 12.

This “improved process” was directly compared with the “original process” which retained medium volume at the maximum working volume, 15 mL, and had a 1 h static period post‐inoculation which was followed by continuous agitation for the duration of the culture (Fig. 3A). Instead of the viable cell density dropping to ∼0.10 × 10^5^ cells/mL after 24 h as was the case with the original process (Fig. 3A), the viable cell density for the improved process increased to 0.36 × 10^5^ cells/mL. This reduced the lag phase by 48 h and resulted in significantly higher cell proliferation rate with a final viable cell density of 2.72 × 10^5^ ± 0.09 cells/mL by 192 h. Compared to the original ambr15 process, the improved process resulted in >70% increase in yield and this increase is reflected in the specific growth rate, cumulative population doublings and doubling times measured (Fig. 3B–D).

### Validation of the “Improved Bioprocess” With the Spinner Flask Culture System

**Figure 4:**
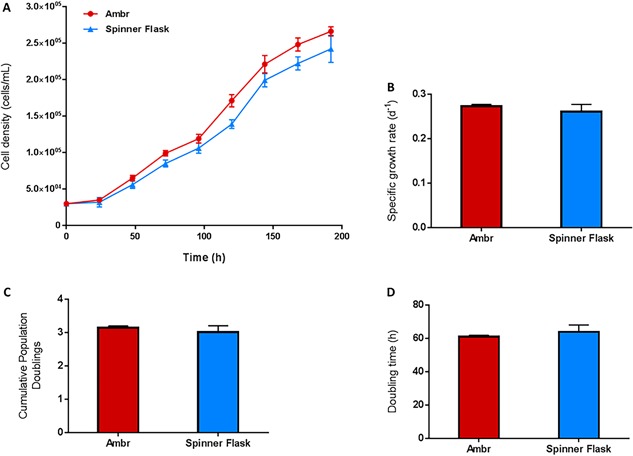
Validation of the improved ambr15 bioprocess with the larger‐scale spinner flask for hMSC donor 1 cells showing (A) the viable cell density for donor 1, (B) specific growth rate, (C) the cumulative population doublings and (D) the doubling time. Data show mean ± SD, *n* = 8.

**Figure 5:**
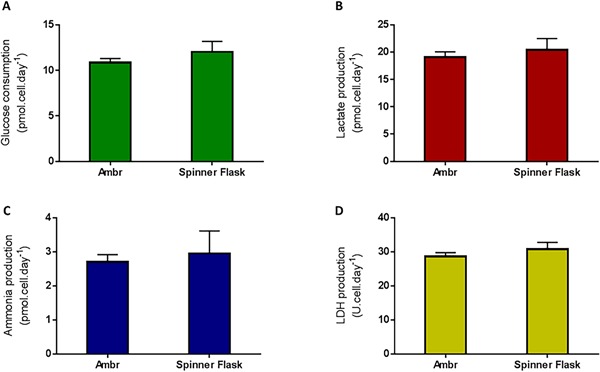
Specific metabolic activity for hMSC donor 1 cells with the ambr15 improved process and validated by the larger‐scale spinner flask. The data show (A) the specific glucose consumption, (B) specific lactate production, (C) the specific ammonia production and (D) the specific LDH production. Data show mean ± SD, *n* = 8.

**Figure 6:**
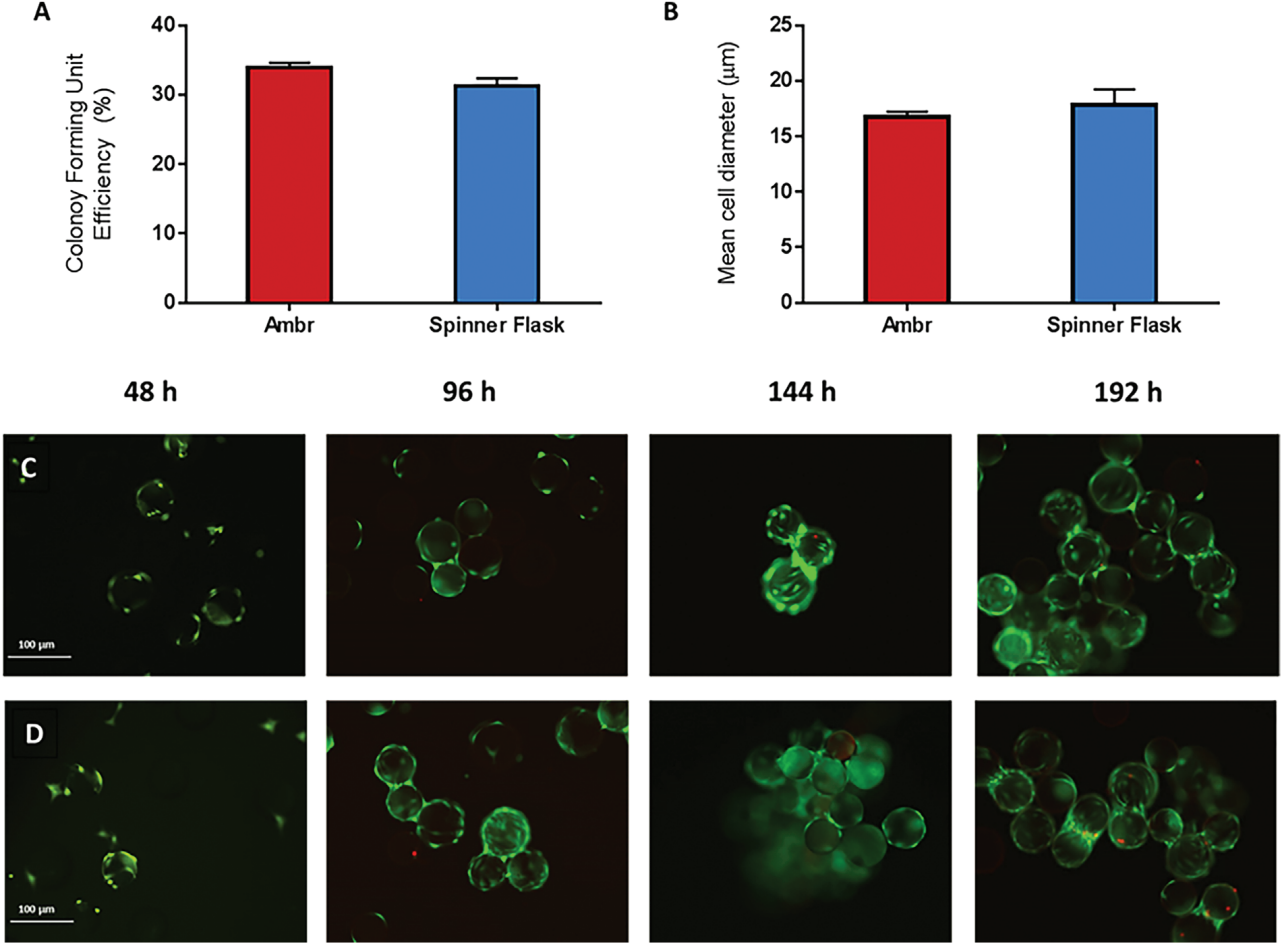
Characterisation of hMSC donor 1 cells cultured with the ambr15 improved process and validated by the larger‐scale spinner flask. The data show (A) colony forming unit efficiency, (B) mean cell diameter, fluorescent staining illustrating viable (green), and non‐viable (red) cells cultured in (C) the ambr15 and (D) spinner flask at different points during culture.

Having developed an improved ambr15 process, similar conditions were employed in the spinner flasks. This change meant using an equivalent microcarrier concentration to medium volume ratio and fill level to the ambr15 and an identical intermittent agitation strategy. Figure 4 shows the culture of hMSCs in both the ambr15 and the spinner flask using the “improved process.” The viable cell densities throughout the culture are almost identical, following the same trend of a reduced lag phase and a final viable cell density of ∼2.5 × 10^5^ cells/mL; the viable cell densities at 192 h of both the ambr15 and the spinner flasks were found not to be significantly different (Fig. 4A). The similarity between the ambr15 and the spinner flask using the improved process was also reflected in the specific growth rates, cumulative population doublings, and doubling times measured (Figs. 4B–D). In addition to the growth kinetics, the metabolic activity of the cells in each culture system was similar; with the specific glucose consumption ∼10 pmol/cell/day and 10.5 pmol/cell/day (Fig. 5A), specific lactate production ∼19 pmol/cell/day and 20 pmol/cell/day (Fig. 5B), specific ammonia production ∼2.8 and 3.0 pmol/cell/day (Fig. 5C) and LDH ∼29 and 30 U/cell/day (Fig. 5D) in the ambr15 and spinner flask, respectively. The similarity between the two systems is further demonstrated by the functionality with respect to colony forming unit efficiency (Fig. 6A) and morphological characteristics of the cells with respect to both mean cell diameter (Fig. 6B) and cell growth on the microcarriers (Fig. 6C and D).

**Figure 7:**
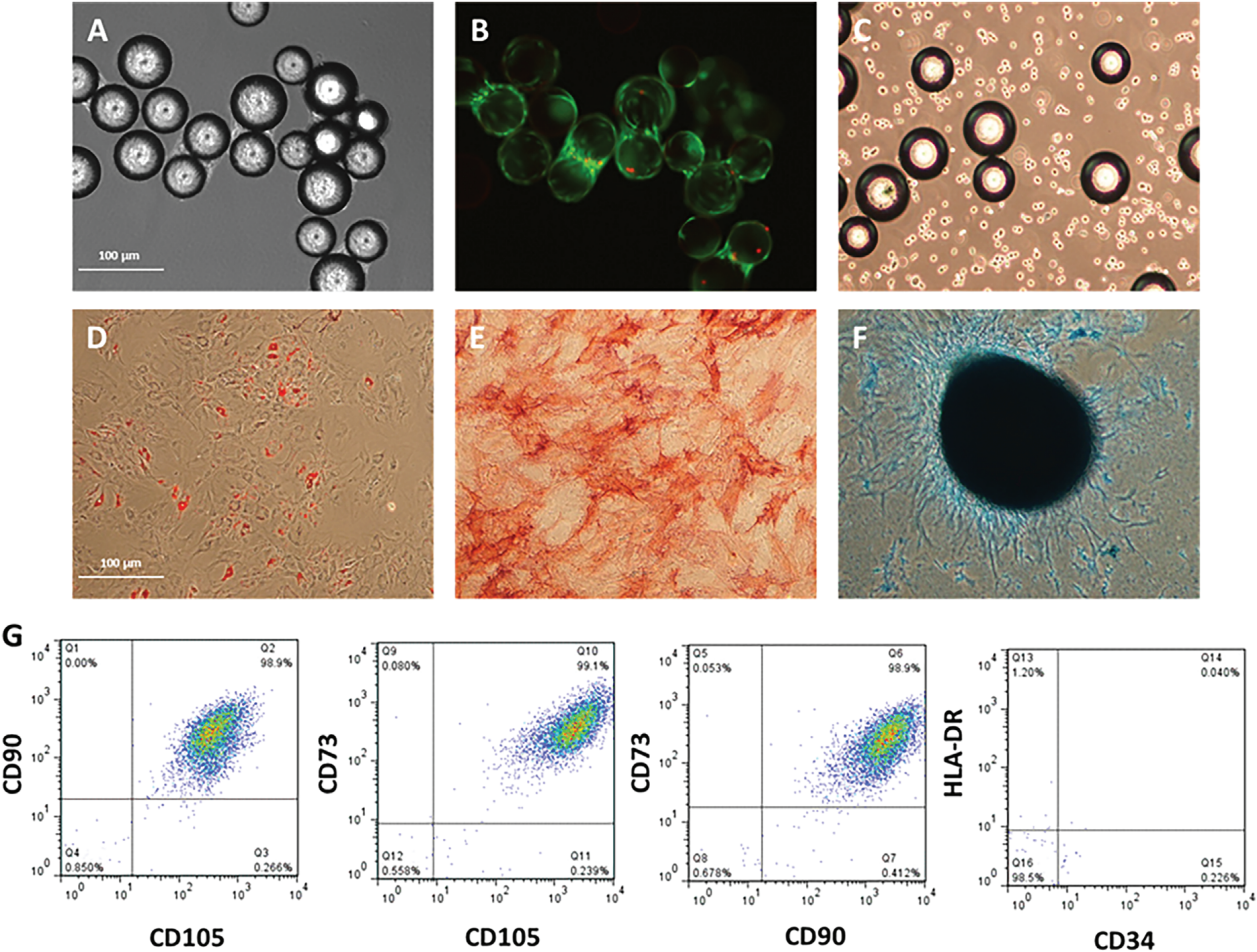
Functional characterization of hMSC donor 1 cells harvested from the improved ambr15 bioprocess. The data show (A) brightfield image of hMSCs growing on Plastic P102‐L microcarriers in the ambr15 microbioreactor vessel, (B) fluorescent staining of same cell‐microcarrier image depicted in (A) with viable (green) and non‐viable (red) cells. (C) Single cells following detachment of hMSCs from the microcarriers using the ambr15 microbioreactor. Tri‐lineage differentiation potential of hMSCs harvested from the ambr15 showing (D) Adipogenic, (E) osteogenic, and (F) chondrogenic differentiation of hMSCs. (G) Multiparameter flow cytometry showing dual gating of CD90, 105, 73, 34, and HLA‐DR for hMSCs post‐harvest from the ambr15 microbioreactor.

Cell identity and quality with respect to the immunophenotypic expression, differentiation, and CFU‐f potential was assessed and cells on microcarriers taken from the ambr15 microbioreactor are shown as brightfield (Fig. 7A) and fluorescent images (Fig. 7B); cells in suspension after detachment are shown in Figure 7C. In addition, hMSCs from both the ambr15 microbioreactor and the spinner flasks that had been harvested at the end of each culture were also assessed in accordance with the ISCT criteria (Dominici et al., 2006), which demonstrated their trilineage differentiation potential toward the osteogenic, chondrogenic, and adipogenic pathways (Fig. 7D–F, respectively for the ambr15) and confirmation of their immunphenotype (Fig. 7G). As in our earlier work, the cells from the spinner flasks also met these criteria (Rafiq et al., 2013a) (data not shown).

**Figure 8:**
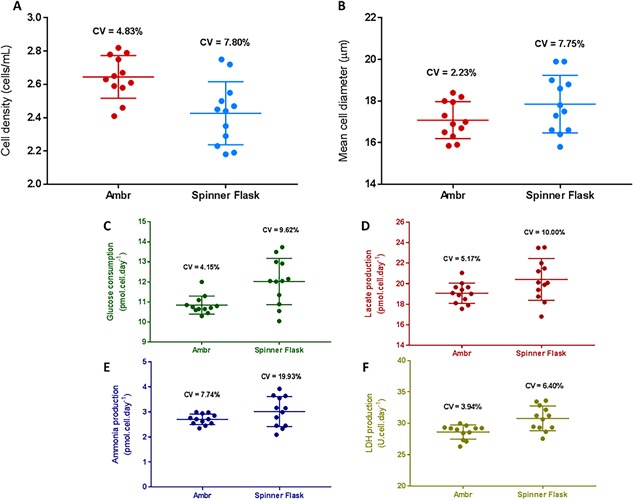
Comparison of extent of variation between the ambr15 and spinner flask for serum‐based hMSC donor 1 microcarrier culture with data showing (A) viable cell density, (B) mean cell diameter, (C) specific glucose consumption, (D) specific lactate production, (E) specific ammonia production, and (F) specific LDH production. Data show coefficient of variation (CV), *n* = 12.

Although the growth kinetics and metabolic activity of the cells were similar between the two systems, it was notable that there was a greater level of variation in the spinner flask compared to the ambr15 system across all of the measured parameters including cell density, mean cell diameter and specific metabolite production/consumption (Fig. 8). In all cases, the coefficient of variation (CV) was lower using the ambr15 system compared to the spinner flasks, demonstrating that the ambr15 was a more consistent system for the growth of hMSCs on microcarriers (Fig. 8), presumably because of the tighter control of pH and dO_2_.

### Serum‐Free Expansion of hMSCs in ambr15 and Spinner Flasks

**Figure 9:**
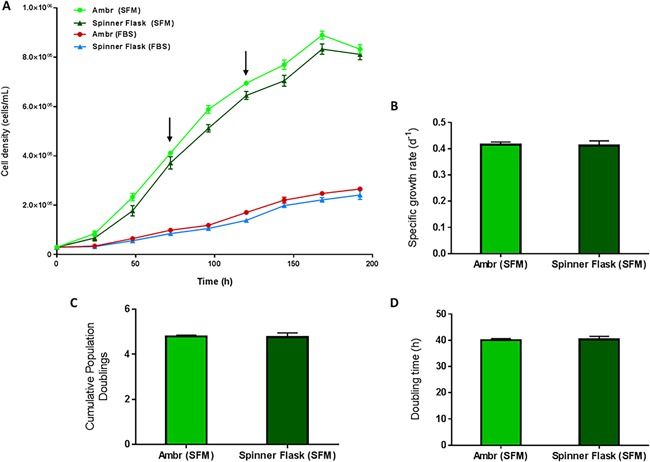
Growth kinetics of hMSC donor 1 cells for serum‐free (SFM) and fetal bovine serum (FBS)‐based media in both the ambr15 and spinner flasks with data showing (A) the viable cell density, (B) specific growth rate, (C) the cumulative population doublings, and (D) the doubling time. Data show mean ± SD, *n* = 12. The arrows in (A) indicate the point at which additional microcarriers were added to the culture for both the SFM and FBS processes.

The process was then adapted to culture the hMSCs in serum‐free medium. Figure 9 illustrates a significant increase in yield obtained with both the ambr15 and the spinner flask when using the serum‐free medium. The final viable cell density obtained with both the ambr15 and spinner flask in the serum‐free medium was >8.1 × 10^5^ cells/mL while the equivalent serum‐based medium studies yield was ∼2.2 × 10^5^ cells/mL, resulting in >250% increase in yield (Fig. 9A). To accommodate, the increase in growth and requirement for surface area, additional microcarrier surface area was provided at 72 h and 120 h (as indicated by the arrows on Fig. 9A). Additional surface area was aseptically transferred to the culture to obtain a mean ratio of 50 cells/microcarrier. The amount added was calculated from the cell number obtained from sampling each vessel and the number of beads in the culture at the time of addition. Though there were distinct differences in performance with the different media, as with the study described in Validation of the “Improved Bioprocess” With the Spinner Flask Culture System section, the growth kinetics in the ambr15 and the spinner flask were similar in both media; as represented by the viable cell densities (Fig. 9A), the specific growth rates (Fig. 9B), the cumulative population doublings (Fig. 9C) and the doubling time (Fig. 9D). Likewise, when the study was repeated with an additional BM‐hMSC donor, the growth kinetics between the ambr15 and the spinner flasks were similar (Supplementary Fig. S1). The overall cell yield between the two donors was different; however such differences between the two donors were also exhibited in monolayer cultures (data not shown) and is attributed to donor‐to‐donor variation.

Metabolite analysis of the serum‐based and serum‐free medium expansion demonstrated a decrease in the glucose concentration, particularly toward the latter stages of the cultures which may have contributed to the decrease in cell concentration in SFM conditions (Supplementary Fig. S2). There is a concomitant increase in the concentration of both lactate and ammonia. However, these do not reach levels which may inhibit cell growth (Schop et al., 2010). The hMSCs cultured in SFM in the ambr15 and spinner flask (data not shown) retained their ability to differentiate toward the adipogenic, ostogenic, and chondrogenic lineages and the expected immunphenotypic expression (Supplementary Fig. S3).

**Figure 10:**
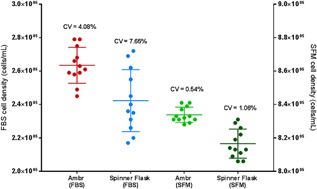
Extent of viable cell density variation in the ambr15 and spinner flask for hMSC donor 1 cells for both serum‐free (SFM) and fetal bovine serum (FBS)‐based cultures. Cell density values for FBS are aligned with the left *y*‐axis and the SFM values with the right *y*‐axis. Data show coefficient of variation (CV), *n* = 12.

When comparing the extent of variation between the two systems (ambr15 and spinner flask) and the media (serum‐based and serum‐free), the ambr15 serum‐free system was the most consistent process with a CV of 0.54% while the equivalent serum‐based process in the ambr15 resulted in a CV of 4.08% (Fig. 10). The process which demonstrated the greatest level of variability was the spinner flask serum‐based process which had a CV of 7.65% and the equivalent serum‐free spinner flask process had a CV of 1.06% (Fig. 10).

## Discussion

With increasing reagent and medium costs and the need for high‐throughput capability, there is a demand for automated systems which can operate at lower working volumes and offer greater levels of parallelization. Clearly, the ambr15 cell culture system potentially provides significant advantages over these other existing small‐scale bioreactor systems suitable for microcarrier culture because of its higher‐throughput capability (up to 48 microbioreactor vessels can be run simultaneously), lower working volume requirements (7–15 mL) and automated liquid handling and culture operation.

Here, we have demonstrated, after some modifications, that the ambr15 microbioreactor can be used successfully for hMSC culture on microcarriers. Importantly, we also demonstrate that it can be used to improve a microcarrier bioprocess. These results were achieved by first improving the mixing and suspension of the microcarriers in the vessel. In detail, this required recognizing the challenges presented by the geometry of the ambr15 vessel (Fig. 1B), in particular the propensity for microcarriers to settle and form aggregates initially under the impeller and then subsequently in the corners of the vessel. The strategy employed involved siliconizing the vessel prior to use, changing the mode of agitation from up‐pumping to down‐pumping and increasing the agitation speed (from 300 to 400 rpm) to ensure full suspension (Nienow, 1997b) during the expansion/culture phase. In addition, attachment of cells to microcarriers was improved by lowering the initial fill volume by 50% and providing an intermittent agitation regime. These process changes have improved our baseline bioreactor process and aligns with process development work conducted by others where such strategies have been employed successfully to improve cell attachment to microcarriers (Carmelo et al., 2015; Frauenschuh et al., 2007; Yuan et al., 2012). In addition to ensuring full suspension, these changes limited the extent of clumping around the base and corners of the vessel and together with the increase in agitator speed during harvesting (from 650–800 rpm), ensured that as many cells as possible (>95%) were harvested.

Collignon et al. (2010) specified criteria for impeller selection specifically for microcarrier culture which included: (1) maintaining microcarriers in complete suspension; and (2) effective homogeneity of culture medium. The down‐pumping mode is generally preferred for solid particle suspension including microcarriers (Ibrahim and Nienow, 2004) and enables them to be suspended at a lower mean specific energy dissipation rate than other impellers. In addition, they give similar mixing times to other impellers at the same mean specific energy dissipation rates (Nienow, 1997a). Here, this mode effectively ensured that the microcarriers did not settle, clump together or form aggregates on the base and in the corners of the microbioreactor vessel.

There is always concern regarding damage to both free suspension cells and cells on microcarriers in stirred bioreactors and this aspect of the current work has recently been discussed in detail (Nienow et al., 2016) . The usually accepted criterion for damage on microcarriers has been that the size of the Kolmogorov scale of turbulence, λ_K_ should not be less than about 2/3rd the size of microcarrier (Croughan et al., 1987); the microcarriers used in this study (Plastic P102‐L) have a diameter ranging from 112 to 212 μM. In this case, even with a down‐pumping agitator at N_JS_ (=400 rpm), the maximum local specific energy dissipation rate, which defines the minimum Kolmogorov scale was estimated to be 0.142 W/kg to give a Kolmogorov scale (*λ*_K_)_JS_ of 52 μM. Though smaller than the usually accepted scale, the cells were still able to grow to confluence and maintained their quality attributes with respect to differentiation potential, CFU‐f formation, and immunophenotype (Figs. 4, 7 and 9). However, at the speed used for harvesting (800 rpm), the Kolmogorov scale was reduced to 24 μM (Nienow et al., 2014), which is greater than the size of cells once they become detached from the microcarriers during harvesting. In that case, they should not be damaged and once again the cells maintained all their quality attributes (Nienow et al., 2016).

In addition to ensuring adequate suspension of microcarriers, the strategies implemented reduced the amount and extent of clumping (Fig. 2B and C). A large clump such as those observed in Figure 2 would have a severely reduced specific surface area available for mass transfer of nutrients and metabolites to and from the cells, thereby potentially limiting cell growth; and causing possible nutrient/oxygen deprivation at the centre of the clump. Previous work by Jorgensen and Tyers (2004) has demonstrated that cells which experience such deprivation remain in the G1 phase of the cell cycle for longer, thus resulting in a decrease in proliferation potential. In addition, Ferrari et al. (2012) indicate the detrimental effect of cell aggregation due to the reduced proliferative capacity and highlight the potential for populations with unwanted heterogeneity. They propose a method of avoiding cell aggregation by the inclusion of additional fresh microcarriers during the culture process. This concept is supported by our findings (Fig. 9) and that of Dos Santos et al. (2011b), where the addition of fresh microcarriers to the process was necessary to ensure the continued proliferative capacity of the cells and to avoid clumping or aggregation of cells. The addition of fresh microcarriers spreads the cells more widely on the total increased microcarrier surface available, promoting bead to bead transfer and increasing the area of the cells in direct contact with the medium and available for mass transfer. In addition, it reduces the likelihood of cells forming large cell aggregates or large cell‐microcarrier clumps if the additional microcarriers are provided during the exponential phase of culture, prior to cells reaching confluence.

The improved process that was developed using the ambr15 resulted in improved attachment and a reduction in the lag phase. This improvement was achieved by two key aspects, namely providing only 50% of the final working volume of medium for the first 24 h in conjunction with an intermittent agitation strategy. These strategies resulted in >150% increase in viable cell density after 24 h compared to the original process (no agitation for 24 h and 100% working volume). We posit that these changes had a significant impact on the initial attachment as it exposed the full surface of the microcarrier to the cells and reduced the distance and therefore time taken for the cells to reach the microcarrier surface. The intermittent agitation also resulted in fewer microcarrier‐cell clumps and reduced cell aggregation. Studies have demonstrated that intermittent agitation provides sufficient mass transfer, and prevents the formation of aggregate formation, but more importantly, it also exposes the full surface of the microcarrier to the cells (Carmelo et al., 2015; Frauenschuh et al., 2007; Hervy et al., 2014; Ting et al., 2014; Wang and Ouyang, 1999; Yeatts et al., 2013; Yuan et al., 2012). In addition to improving cell attachment and growth, intermittent agitation can have a favorable effect on functionality. For example, Ting et al. (2014) demonstrate that for human embryonic stem cell (hESC) cultures, intermittent agitation is preferred to both static and continuous agitation and results in a greater cardiomyocyte differentiation efficiency. With respect to the initial medium volume, our findings are similar to those of Dos Santos et al. (2011b), who also found that by adding only 50% of the final working volume during the first 24 h (the attachment period), there was an increase in cell attachment resulting in a reduced lag phase.

Regulatory approval of any therapeutic is dependent, in part, on the development of consistent manufacturing processes (FDA, 2009), and therefore process development efforts should be directed toward minimizing variation. This will also increase cost‐effectiveness of cell‐based therapy manufacture. Our earlier work in T‐flasks (Heathman et al., 2015b) and spinner flasks (Heathman et al., 2015a) showed a significant process improvement with serum‐free medium compared to a serum based medium (>250% increase in yield in the serum‐free medium). Here, the same effect on process yield (Fig. 9) and consistency (Fig. 10) has been demonstrated in the ambr15. Previous studies have also demonstrated that process automation can improve consistency for both hMSCs and hESCs, but this work has been predominantly limited to monolayer culture (Thomas et al., 2007, 2009). A similar improvement has been shown here with the better control provided by the ambr15 improving process consistency compared to spinner flasks in both serum containing (Fig. 8) and serum‐free media (Fig. 10).

Clearly, both changes are important and Figure 10 gives some indication of the synergy from using both. For example, while the use of the automated ambr15 systems, with its improved control compared to the spinner flask, reduces the CV on cell density in the serum containing medium from 7.65% to 4.08%, the switch to serum free reduces these two values to 1.06% and 0.54%, respectively; a total of 12 replicates were used to calculate the CV. Thus the combination of both serum‐free and automated processing improves the consistency more than 10‐fold. This improvement has clear advantages for both autologous and allogeneic bioprocess development with reduced costs and improved efficiency. Additionally, if processes can be effectively scaled down, this enables larger amounts of data to be collected during development, thereby improving process understanding which facilitates validating future comparability studies if process changes need to be made. If the data collection and analysis of development efforts can be automated, this will lead to significant time and cost savings and will reduce errors. Moreover, the significant increase in yield associated with serum‐free expansion reduces the process time required to achieve a particular batch size, which should also reduce overall process cost. Although not widely explored in the literature, it is thought that the use of a serum‐free medium improves yield and consistency in comparison to serum‐based cultures due to the activation of specific growth factors in the former, in particular platelet‐derived growth factors (Heathman et al., 2015b; Veevers‐Lowe et al., 2011).

Although this study focused on culture parameters such as intermittent agitation and initial medium volume, the major utility of the ambr15 microbioreactor system is its use as an effective high‐throughput process development platform. This feature will enable the study of other key culture parameters such as pH, dO_2_ concentration, growth media, dissociation reagents and cell, and microcarrier inoculation density among others, thereby expediting adherent cell/microcarrier process development.

## Conclusions

Microbioreactors play an important role in process development of therapeutics including cell‐based therapies by reducing the cost and time associated with development. This study describes the approach taken to make the ambr15 microbioreactor amenable for hMSC microcarrier culture and the subsequent bioprocess development using the system. The improved process developed using the ambr15, which included an intermittent agitation strategy and 50% final working volume for the first 24 h, significantly increased the yield and was subsequently validated using a larger‐scale spinner flask culture. Moving toward serum‐free expansion using the improved process increased the yield by >250% in comparison to serum‐based culture and, in conjunction with the automated capability of the ambr, improved process consistency 10‐fold compared to manually‐operated spinner flask serum‐based culture. Cell identity and quality with respect to immunphenotypic expression, multipotency and CFU‐f potential was retained with the improved process, although impact on specific CQAs for different cell therapy products would need to be addressed depending on the intended clinical indication. Nonetheless, this study demonstrates that the ambr15 microbioreactor system is an effective tool for bioprocess development and optimization of hMSC microcarrier culture processes.

This study has been funded by the Engineering and Physical Sciences Research Council via the E‐TERM Landscape Fellowship programme (grant no. EP/I017801/1).

## Supporting information

Additional supporting information may be found in the online version of this article at the publisher's web‐site

**Figure S1**. Growth kinetics of hMSCs donor 2 cells using serum‐free (SFM) and fetal bovine serum (FBS)‐based media in both the ambr15 and spinner flasks with data showing the viable cell density.

**Figure S2**. Nutrient and metabolite flux for hMSC donor 1 cells expanded on microcarriers in the serum‐based and serum‐free cultures in both the ambr and spinner flasks.

**Figure S3**. Functional characterisation of hMSCs from donor 1 harvested from the serum‐free ambr15 bioprocess.
