# Supplementary figures and images for: Process development of human multipotent stromal cell microcarrier culture using an automated high‐throughput microbioreactor
Source: Biotechnol Bioeng. 2017 Jul 27;114(10):2253–66. doi: 10.1002/bit.26359 (PMC5615370; doi:10.1002/bit.26359)

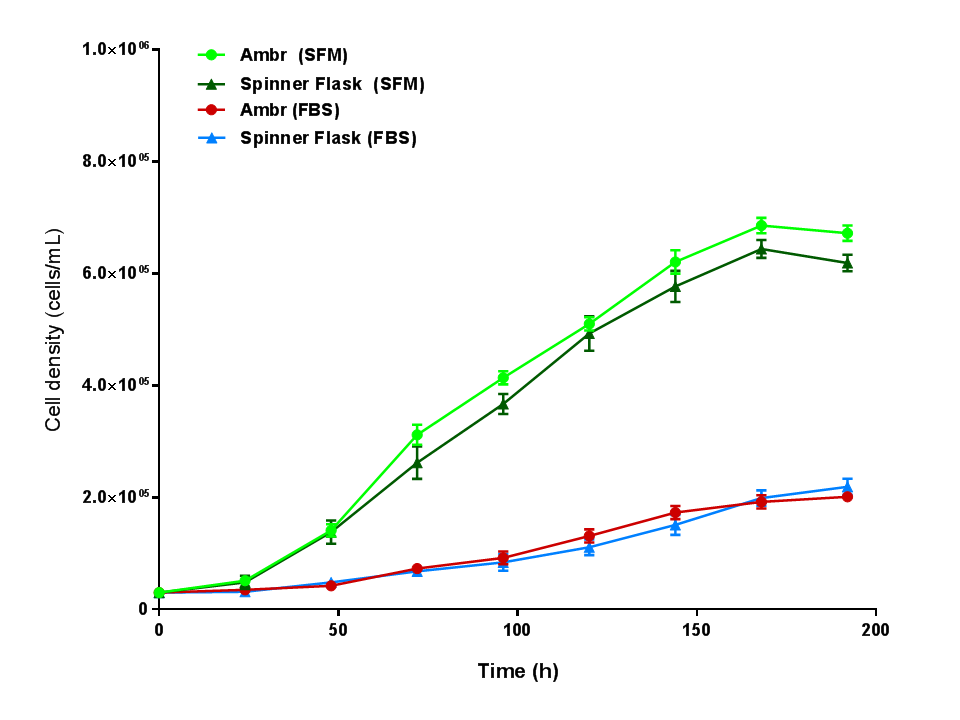

Supplement: Supplementary file 1 — Figure S1. Growth kinetics of hMSCs donor 2 cells using serum‐free (SFM) and fetal bovine serum (FBS)‐based media in both the ambr15 and spinner flasks with data showing the viable cell density. [file BIT-114-2253-s001.tif]

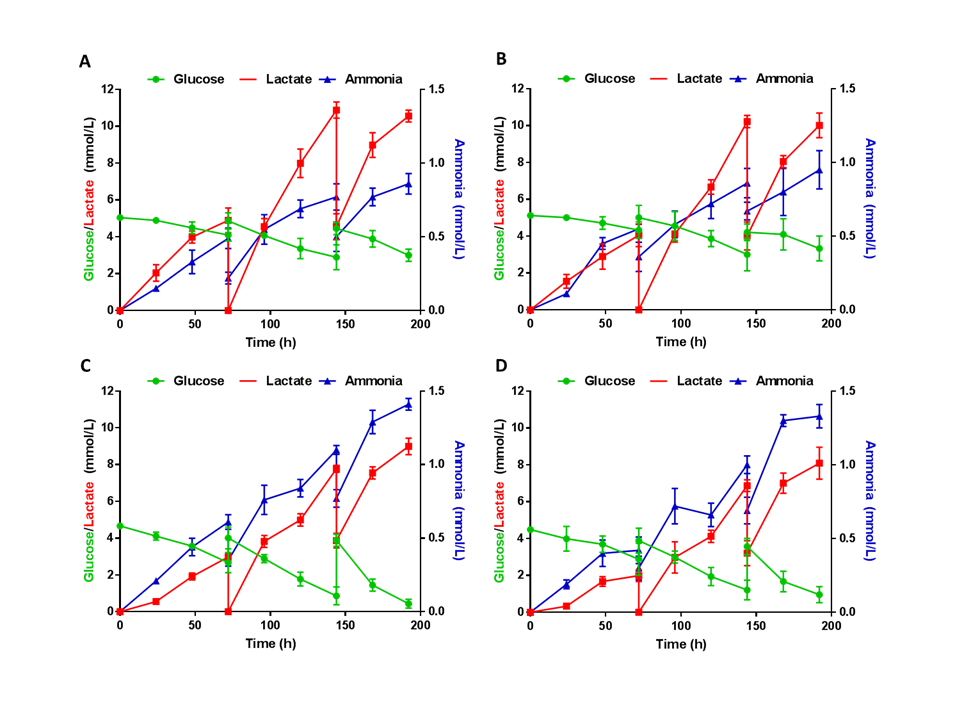

Supplement: Supplementary file 2 — Figure S2. Nutrient and metabolite flux for hMSC donor 1 cells expanded on microcarriers in the serum‐based and serum‐free cultures in both the ambr and spinner flasks. [file BIT-114-2253-s002.tif]

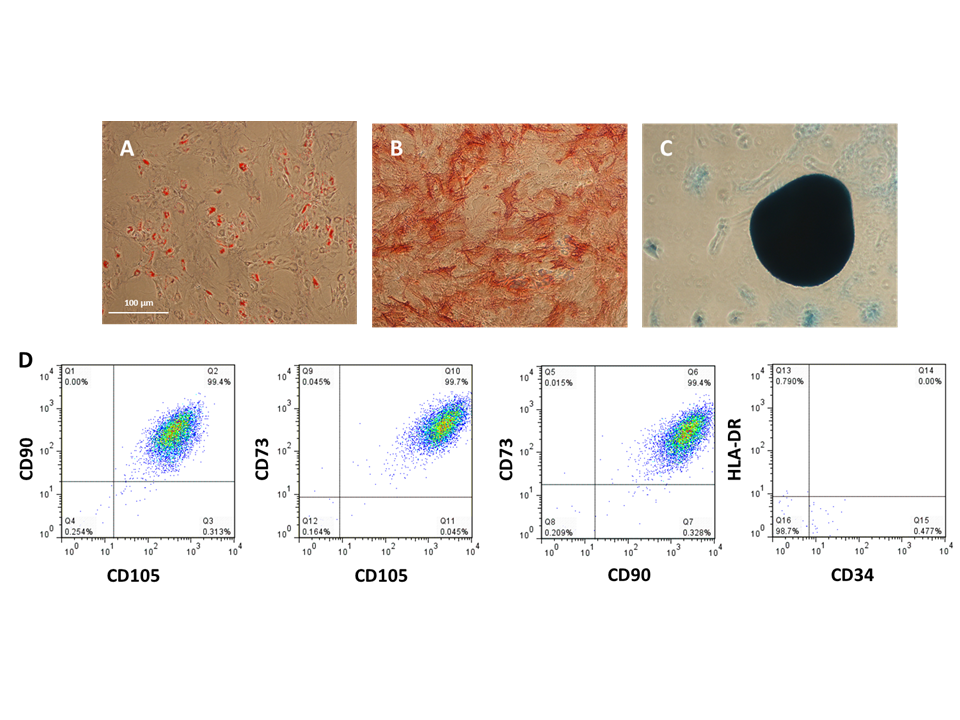

Supplement: Supplementary file 3 — Figure S3. Functional characterisation of hMSCs from donor 1 harvested from the serum‐free ambr15 bioprocess. [file BIT-114-2253-s003.tif]
